# Halogenated indoles selectively modulate plant growth and root development

**DOI:** 10.3389/fpls.2026.1826917

**Published:** 2026-09-15

**Authors:** Hyeonwoo Jeong, Kwang-Hyun Baek, Jin-Hyung Lee, Jintae Lee

**Affiliations:** 1 School of Chemical Engineering, Yeungnam University, Gyeongsan, Republic of Korea; 2 Department of Biotechnology, Yeungnam University, Gyeongsan, Republic of Korea

**Keywords:** auxin, halogenated indoles, indole, plant growth modulation, structure-activity relationship

## Abstract

Indole is a central scaffold in microbiology and also functions as an auxin precursor and signaling molecule in plants. However, the growth-modulating potential of substituted indoles remains largely unexplored. Here, we screened 87 indole and indole-derived compounds for effects on early seedling growth in two *Brassicaceae* species, radish and Chinese cabbage. Seed germination was largely unaffected, whereas marked variability was observed in total growth and particularly in root development. The parent compound indole strongly promoted seedling growth to approximately 140% relative to the control, while classical auxin-related derivatives, including indole-3-acetic acid and indole-3-acetonitrile, suppressed growth to approximately 31% of the untreated control at 10 μg/mL. Several substituted indoles exhibited enhanced growth, with di-halogenated derivatives such as 7-chloro-5-fluoroindole producing the strongest stimulation, with total seedling length and root growth reaching approximately 155% and 220% of the untreated control, respectively. Structure–activity relationship analysis revealed clear position- and halogen-dependent trends, with substitutions at C6–C7 and specific di-halogenation patterns associated with root-biased promotion, whereas auxin-like side chains and small halogens at C4–C5 were generally unfavorable. These findings identify halogenated indoles as a tunable chemotype capable of modulating plant growth and root development.

## 1 Introduction

Plant growth and development are regulated by interconnected hormonal and metabolic networks that integrate endogenous and environmental signals. Among these regulators, auxins play central roles in cell elongation, organogenesis, tropic responses, and root system architecture, thereby directly influencing nutrient acquisition and overall plant performance. Indole-3-acetic acid (IAA) is the predominant endogenous auxin in higher plants, and its biosynthesis, transport, and signaling are tightly regulated to produce spatially and temporally defined developmental outputs (Vanneste and Friml, 2009; Zhao, 2010). However, exogenous auxin application often produces concentration-dependent and context-specific outcomes, including growth inhibition at elevated levels. In roots, external auxins can suppress primary root elongation while promoting lateral root formation, reflecting the complex relationship between auxin dose, transport, and tissue sensitivity (Overvoorde et al., 2010; Roychoudhry and Kepinski, 2022). These dose-dependent responses highlight how small-molecule perturbations can lead to either growth promotion or suppression depending on concentration, species, and developmental stage (Zhao, 2010).

Chemical modification of the auxin scaffold demonstrates that small structural changes in indole chemistry can markedly alter biological activity. An example is the naturally occurring halogenated auxin 4-chloroindole-3-acetic acid (4-Cl-IAA), which accumulates in developing seeds of several legumes and is considered one of the most potent naturally occurring auxins (Tivendale et al., 2012). Biosynthetic studies in pea have shown that 4-Cl-IAA is produced from chlorinated tryptophan intermediates, indicating that plants can generate and metabolize halogenated indole derivatives within hormone pathways (Tivendale et al., 2012). Comparative studies of IAA and 4-Cl-IAA further indicate that chlorination can enhance potency and modify physiological responses, likely through altered metabolism and signaling dynamics (Karcz and Burdach, 2002). In addition, metabolic engineering and feeding studies in *Arabidopsis thaliana* have demonstrated the formation and conjugation of chlorinated indole metabolites, supporting the view that halogenation can serve as a powerful chemical handle to tune indole-associated plant growth (Walter et al., 2020).

Beyond auxin biology, the indole nucleus itself occupies a prominent role in plant and rhizosphere chemistry. Indole is produced in diverse ecological contexts and functions as an interspecies and interkingdom signaling molecule involved in plant growth, defense, and communication (Lee et al., 2015). In maize, herbivory triggers rapid indole emission that primes systemic tissues and neighboring plants for enhanced defense through jasmonate-related signaling (Erb et al., 2015; Hu et al., 2019; Sorg et al., 2025). Similar defense-priming effects have been reported in rice and tea, where indole exposure enhances early signaling responses and stress-associated pathways (Sun et al., 2022). In addition to its role in defense, indole also functions as a plant growth modulator in plant–microbe communication. Volatile indole produced by rhizobacteria can stimulate seedling growth and root development, partly by interfering with auxin signaling and polar auxin transport, thereby reshaping root system architecture (Bailly et al., 2014). Although no specific indole receptor has yet been identified in plants, indole and its derivatives are recognized as ligands of the aryl hydrocarbon receptor (AhR)—a cytosolic, ligand-activated transcription factor characterized in animal systems (Dvorak et al., 2021; Hubbard et al., 2015; Sorg et al., 2025; Sun et al., 2022; Vyhlidalova et al., 2020). These findings position indole as a non-canonical regulator capable of influencing plant growth through hormone-network interactions rather than acting solely as an auxin precursor.

Although the roles of indole and indole-derived metabolites in plant growth regulation and stress responses are increasingly recognized, most studies have focused on canonical auxins, indoleamines, or volatile signaling molecules (Sun et al., 2022). In contrast, halogenated indoles lacking the classical auxin side chain—that is, substituted indoles structurally distinct from indole-3-acetic acids—have received little systematic investigation in plant systems. Moreover, whether halogenated indoles exert beneficial, neutral, or inhibitory effects on plant growth has not been systematically investigated, leaving their biological significance in plant systems largely unknown. Given that halogen substitution can dramatically alter auxin activity and that indole itself can modulate plant growth, halogenation of the indole scaffold may represent an underexplored strategy for tuning plant growth responses independently of classical auxin signaling.

Here, we address this gap by conducting a systematic phenotypic screen of indole and a broad panel of substituted indole derivatives, including mono- and di-halogenated indoles, to evaluate their ability to modulate early seedling growth and root development. Growth responses were assessed in two *Brassicaceae* crops (radish and Chinese cabbage). We further performed concentration–response validation for representative growth-promoting and growth-suppressing indole scaffolds to define activity windows and establish robust phenotypic trends. Structure–activity relationship (SAR) analysis was performed to understand the roles of halogens (F, Cl, Br, I) at specific positions on the indole ring. This study identifies halogenated indoles as promising modulators of early seedling growth and root development, demonstrates that their biological activity depends on the identity and position of halogen substitution, and provides the first systematic evidence for the biological significance of halogenated indoles in plant growth, thereby establishing a foundation for future mechanistic studies and agricultural applications.

## 2 Materials and methods

### 2.1 Chemicals and plants

Radish seeds were obtained from Segye Seed Co. (Gwangju, Korea), whereas Chinese cabbage seeds were sourced from Asia Seed Co. (Seoul, Korea). Eighty-seven indole derivatives (purity >95%, **Supplementary Table 1**) were acquired from Combi-Blocks (San Diego, CA, USA). Each compound was dissolved in dimethyl sulfoxide (DMSO) to make a 100 mg/mL stock solution. The prepared stocks were divided into single-use aliquots and kept at −20 °C until use. Prior to each experiment, the stock solutions were diluted to the desired working concentrations. All compounds were initially screened at 10 μg/mL, whereas indole, indole-3-acetic acid (IAA), indole-3-acetonitrile (IAN), and 7-chloro-5-fluoroindole were additionally evaluated at 5, 10, 20, and 100 μg/mL. For all treatments, the final DMSO concentration was adjusted to remain below 0.1% (v/v) in order to avoid solvent interference. Among the screened library, indole, indole-3-acetic acid (IAA), indole-3-acetonitrile (IAN), and 7-chloro-5-fluoroindole were selected for subsequent comparative analyses. IAA and IAN were included as representative auxin-related reference compounds, indole as the parent compound, and 7-chloro-5-fluoroindole because it exhibited the strongest growth-promoting activity in the initial screening.

### 2.2 Seed assay

The effects of indole derivatives on early seedling growth were evaluated using radish (*Raphanus sativus* L.) and Chinese cabbage (*Brassica rapa* L.). Uniform, healthy seeds of similar size and appearance were selected for all experiments. Seeds were rinsed five times with sterile distilled water and subsequently soaked in sterile distilled water for 3 h. After soaking, the seeds were air-dried under sterile conditions. Seeds were cultured on Murashige and Skoog (MS) agar medium (0.86 g/L MS salts, 0.7% agar). The medium was autoclaved at 121 °C for 16 min, cooled to room temperature, and then supplemented with the respective compounds to achieve the desired final concentrations. The study consisted of two sequential experiments. In the first experiment, all 87 indole derivatives were screened at 10 μg/mL using radish seedlings. In the second experiment, indole, indole-3-acetic acid (IAA), indole-3-acetonitrile (IAN), and 7-chloro-5-fluoroindole were evaluated at 0, 5, 10, 20, and 100 μg/mL in both radish and Chinese cabbage. Plates without compound treatment were used as negative controls. Ten seeds were placed on each plate. For the initial screening experiment, two independent plates were prepared for each treatment. For the concentration-dependent experiments using radish and Chinese cabbage, two independent plates were prepared for each concentration, and the entire experiment was repeated twice. For quantitative analysis, the largest and the smallest seedlings on each plate were excluded, and the remaining eight seedlings were used for growth measurements. The plates were incubated at 20 °C for 5 days under a 12 h light/12 h dark photoperiod. No additional humidification was provided during the incubation period because the MS agar medium maintained sufficient moisture throughout the experiment. Germination rates were recorded daily, and seedling lengths were measured on day 5.

### 2.3 Structure–activity relationship analysis

Indole-based library consisting of 87 compounds (**Figure 1**; **Supplementary Table 1**) was used for screening at a concentration of 10 μg/ml. This concentration was selected based on preliminary experiments and our previous study (Jeong et al., 2025), because it provided clear discrimination of growth responses among indole derivatives and enabled the identification of compounds with robust growth-modulating activity under relatively stringent screening conditions. According to the substitution pattern positions (C2–C7) on the indole ring and the types of substituents, structure–activity relationship (SAR) analysis for plant growth was performed. For each treatment, values measured from 8 individual seedlings were averaged and normalized to the untreated control within each experiment (control = 100%). Root growth rate was used as the primary activity indicator for SAR interpretation, and total growth rate was used as a secondary indicator. Strong promoters were classified as ≥130%, moderate promoters as 129–115%, neutral as 114–90%, and inhibitors as ≤90%. To identify favorable substitution motifs, growth responses were analyzed according to substitution positions and halogen types (F, Cl, Br, I). For di-halogenated indoles, substitution patterns were evaluated as position pairs (e.g., C4/C6, C5/C7). When compounds with the corresponding substitutions consistently showed growth-promoting effects in radish seedlings, the motif was considered favorable.

**Figure 1:**
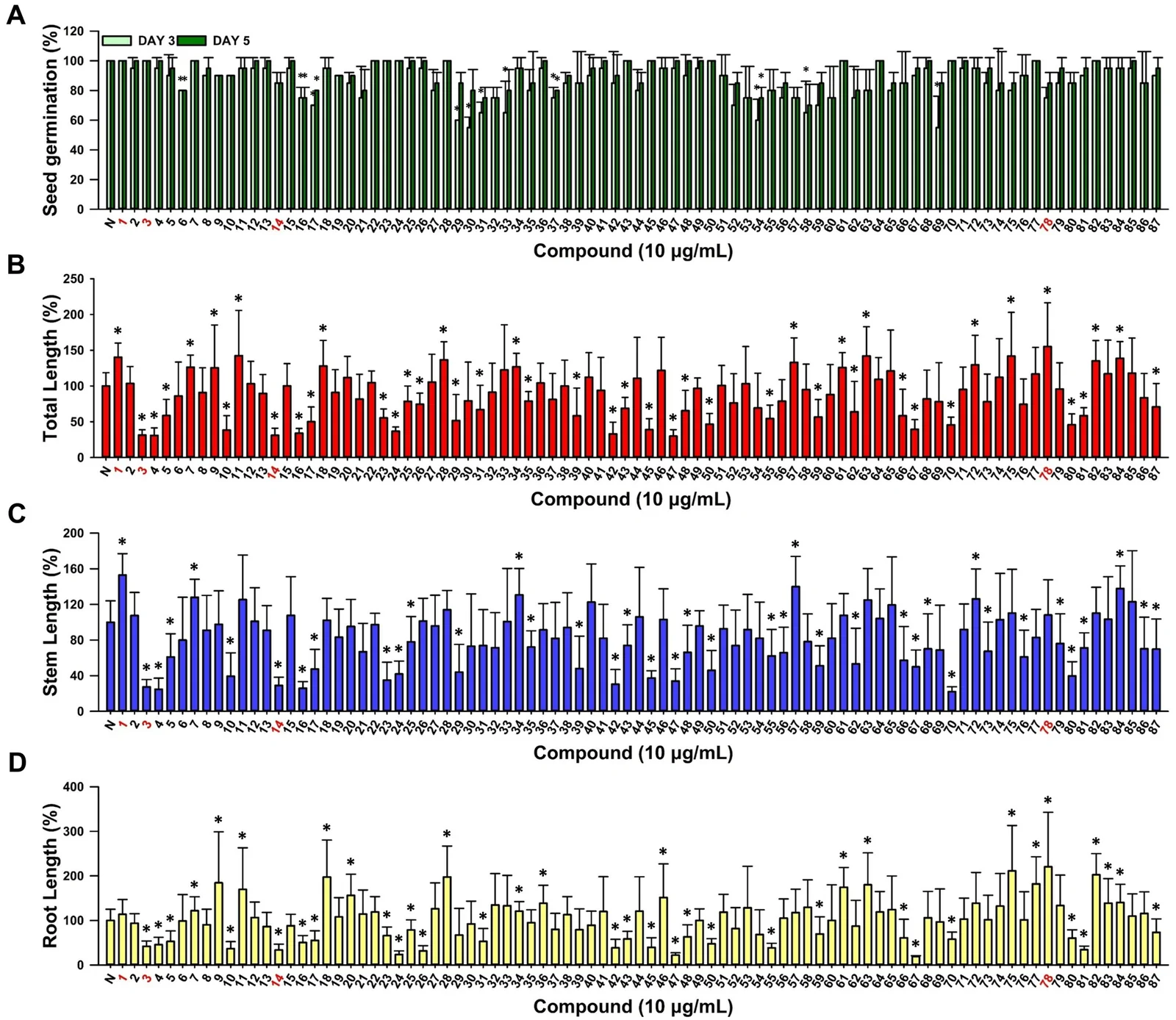
Global screening of indole and substituted indole derivatives for modulation of seedling growth of radish. Bar plots summarizing the relative effects of 87 indole-based compounds at 10 μg/mL on early seedling development compared with untreated controls (set to 100%) for *R. sativus*. Seed germination rate **(A)**, total seedling length **(B)**, stem length **(C)**, and root length **(D)** after 5 days. Data represent mean values obtained from two independent plates, with eight seedlings analyzed per plate after excluding the largest and the smallest seedlings. Compound numbers correspond to the following representatives in red: #1, indole; #3, indole-3-acetic acid (IAA); #14, 3-indoleacetonitrile (IAN); #78, 7-chloro-5-fluoroindole. N denotes the untreated control. *p < 0.05 compared with untreated control. Compound numbers together with chemical names and structures are provided in **Supplementary Table 1**.

### 2.4 Statistical analyses

All experiments were conducted with a minimum of eight individual seedlings per treatment group. Data are presented as mean ± standard deviation (SD). Statistical significance among multiple groups was evaluated using one-way analysis of variance (ANOVA) followed by Dunnett’s multiple comparisons test. A p value of ≤ 0.05 was considered statistically significant.

## 3 Results

### 3.1 Screening of substituted indoles identifies multiple growth-promoting scaffolds

To evaluate the growth-modulating activity of indole scaffolds, a total of 87 indole and indole-derived compounds were screened at 10 μg/mL using seedling assays in radish (**Supplementary Table 1**). Growth responses were quantified by measuring seed germination, total seedling length, stem length, and root length relative to untreated controls (**Figure 1**). Across the library, seed germination was largely unaffected (**Figure 1A**), whereas pronounced variability was observed in total growth and, most notably, root development (**Figures 1B–D**). These results indicate that indole derivatives primarily modulated post-germination growth processes rather than affecting seed viability.

Unless otherwise indicated, all percentage values in this section are expressed relative to the untreated control (control = 100%). The parent compound indole (#1) at 10 μg/mL showed one of the strongest overall growth-promoting effects, with total growth of approximately 140%, accompanied by marked enhancement of stem growth (~153%) and moderate stimulation of root elongation (~114%). In contrast, several classical auxin-related indole derivatives showed clear growth suppression under the same conditions. For example, indole-3-acetic acid (IAA, #3) at 10 μg/mL reduced total growth to ~31% and root growth to ~42%, while natural 3-indoleacetonitrile (IAN, #14) similarly decreased total growth to ~31% and root growth to ~34% (**Supplementary Table 1**). These results indicate that, within this assay window, auxin-type side chains are associated with growth inhibition rather than promotion during early seedling development.

A broad range of substituted indoles exhibited intermediate to strong growth-promoting activity. Several compounds at 10 μg/mL showed total growth above 120%, including 2-methylindole (~125%), 2-methylindole-3-carboxaldehyde (~142%), and multiple halogenated derivatives such as 6-iodoindole (~130%), 4,6-dibromoindole (~137%), and 7-iodoindole (~135%) (**Supplementary Table 1**). In many cases, growth promotion was primarily driven by root elongation rather than stem extension. For example, 2-methylindole increased root growth to ~185%, and 4-bromo-6-chloroindole and 4,6-dibromoindole enhanced root growth to nearly ~197%.

Among all tested compounds, the strongest phenotype was observed with selected di-halogenated indoles. 7-Chloro-5-fluoroindole (#78) produced the highest total growth (~155%), accompanied by a striking increase in root length (~220%). This magnitude of root enhancement substantially exceeded that observed with the parent scaffold and most mono-halogenated analogues. Similarly, 7-bromo-5-fluoroindole (~142% total; root ~211%) and 6-bromo-5-fluoroindole (~142% total; root ~180%) showed strong root-dominant growth enhancement. These findings indicate that specific halogenation patterns can substantially amplify the growth-modulating activity of the indole scaffold, particularly in the root system.

In contrast, several mono-halogenated indoles with fluorine or chlorine substitutions at proximal ring positions showed reduced growth. For instance, 4-fluoroindole (~37% total), 5-fluoroindole (~33% total), and 6-chloroindole (~39% total) were consistently associated with suppressed total and root growth. These results suggest that both the position and identity of the halogen strongly influence biological activity.

Overall, the global screen revealed that indole derivatives span a broad spectrum from strong growth suppression to pronounced growth promotion. While auxin-type indole derivatives tended to inhibit seedling growth at 10 μg/mL, several non-auxin indoles—particularly those bearing halogen substitutions at specific ring positions—showed strong growth-promoting activity, with the most pronounced effects observed in root development.

### 3.2 Growth modulation is consistently observed in two Brassicaceae species

To determine whether the growth-modulating effects observed in the initial screen were species-specific, four representative compounds (0, 5, 10, 20, and 100 μg/mL) were further evaluated in two *Brassicaceae* plants, radish and Chinese cabbage. Visual inspection and quantitative measurements revealed that the overall response patterns were highly consistent between the two species, particularly for compounds showing strong growth-promoting or growth-suppressing activity (**Figure 2**).

**Figure 2:**
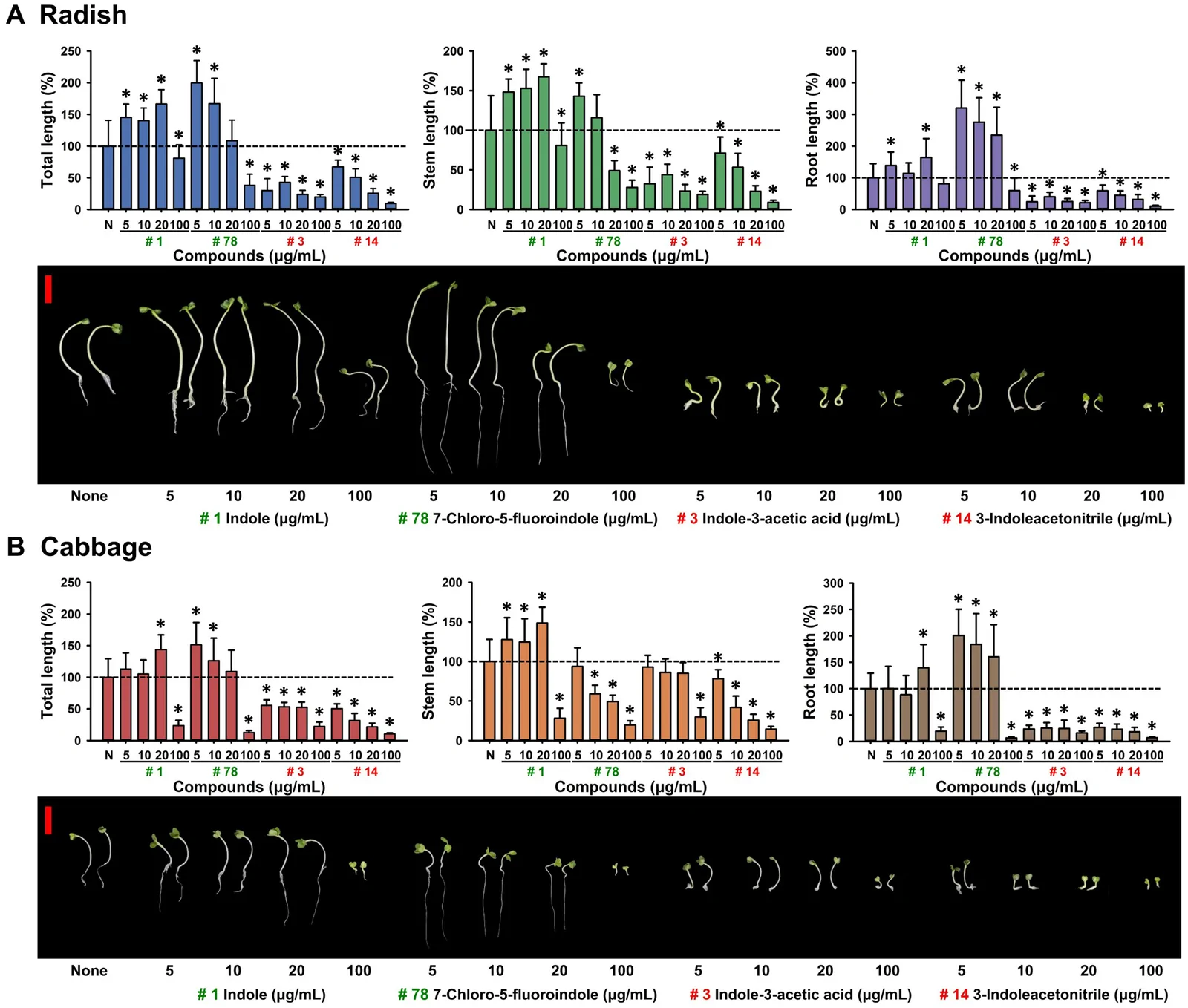
Growth modulation by representative indole derivatives in two *Brassicaceae* species. Radish **(A)** and Chinese cabbage **(B)** seedlings treated with selected indole derivatives showing representative growth-promoting and growth-suppressing phenotypes. Seedlings were exposed to indole (#1), indole-3-acetic acid (IAA, #3), 3-indoleacetonitrile (IAN, #14), and 7-chloro-5-fluoroindole (#78), and growth was monitored for 5 days. N denotes the untreated control. The red scale bar represents 2 cm. Data represent mean values from eight biological replicates per treatment. *p < 0.05 compared with untreated control.

In both radish and Chinese cabbage, the parent scaffold indole produced a clear growth-promoting phenotype, characterized by increased seedling length and enhanced stem length compared with untreated controls (**Figures 2A, B**). Similarly, the synthetic derivative 7-chloro-5-fluoroindole, identified as one of the strongest promoters in the primary screen, consistently increased seedling growth in both species, with the most pronounced effects observed in root development. Seedlings treated with indole and 7-chloro-5-fluoroindole showed clear concentration-dependent growth promotion at 5–20 μg/mL, with the strongest effects observed in this intermediate range. At higher concentrations, both compounds showed a gradual decline in growth, indicating a dose-dependent shift toward reduced stimulation.

In contrast, classical auxin-related indole derivatives showed consistent growth suppression in both plants. Treatment with IAA and IAN resulted in shorter seedlings and reduced root elongation relative to untreated controls in both radish and Chinese cabbage. These inhibitory effects were evident across multiple concentrations and time points, indicating that the suppressive phenotype was not species-specific.

Time-course observations further supported the reproducibility of these responses (data not shown). In both species, growth-promoting compounds led to rapid stem or root elongation, while growth-suppressing compounds consistently produced shorter and less developed growth systems. The similarity in phenotypic patterns between radish and Chinese cabbage suggests that the growth-modulating effects of indole and selected halogenated indoles are reproducible across the two *Brassicaceae* species tested. This cross-species consistency supports the conclusion that substituted indoles, particularly halogenated derivatives, can modulate early seedling growth consistently across the two Brassicaceae species tested rather than producing species-specific effects.

### 3.3 Structure–activity relationship of substituted indoles

A panel of substituted indoles was evaluated for effects on radish seedling growth (**Figure 1**; **Supplementary Table 1**). These results indicate that growth modulation in this series is strongly dependent on substitution pattern and that auxin-like side chains correlate with growth inhibition in this assay window.

Mono-halogen substitution revealed clear position-specific trends (**Figure 3**; **Supplementary Table 1**). In general, substitutions at C4 and C5 were frequently unfavorable, particularly with smaller halogens such as fluorine and chlorine (e.g., 4-fluoro, 4-chloro, and 5-chloro substitutions showed reduced total and root growth). In contrast, C6 and C7 positions were more permissive, especially when substituted with larger halogens. Notably, iodine at C6 showed a clear growth-promoting profile (~130% total; root ~139%), while chlorine at C6 was strongly unfavorable. At C7, larger halogens consistently favored root development, with 7-bromoindole (~117% total; root ~182%) and 7-iodoindole (~135% total; root ~203%) producing pronounced root-biased enhancement. In contrast, fluorine substitution at C7 was generally unfavorable and associated with growth reduction. Collectively, these results suggest that larger halogens (Br, I) at distal ring positions (C6–C7) are favorable, whereas smaller halogens (F, Cl) at proximal positions (C4–C5) are often unfavorable for plant growth.

**Figure 3:**
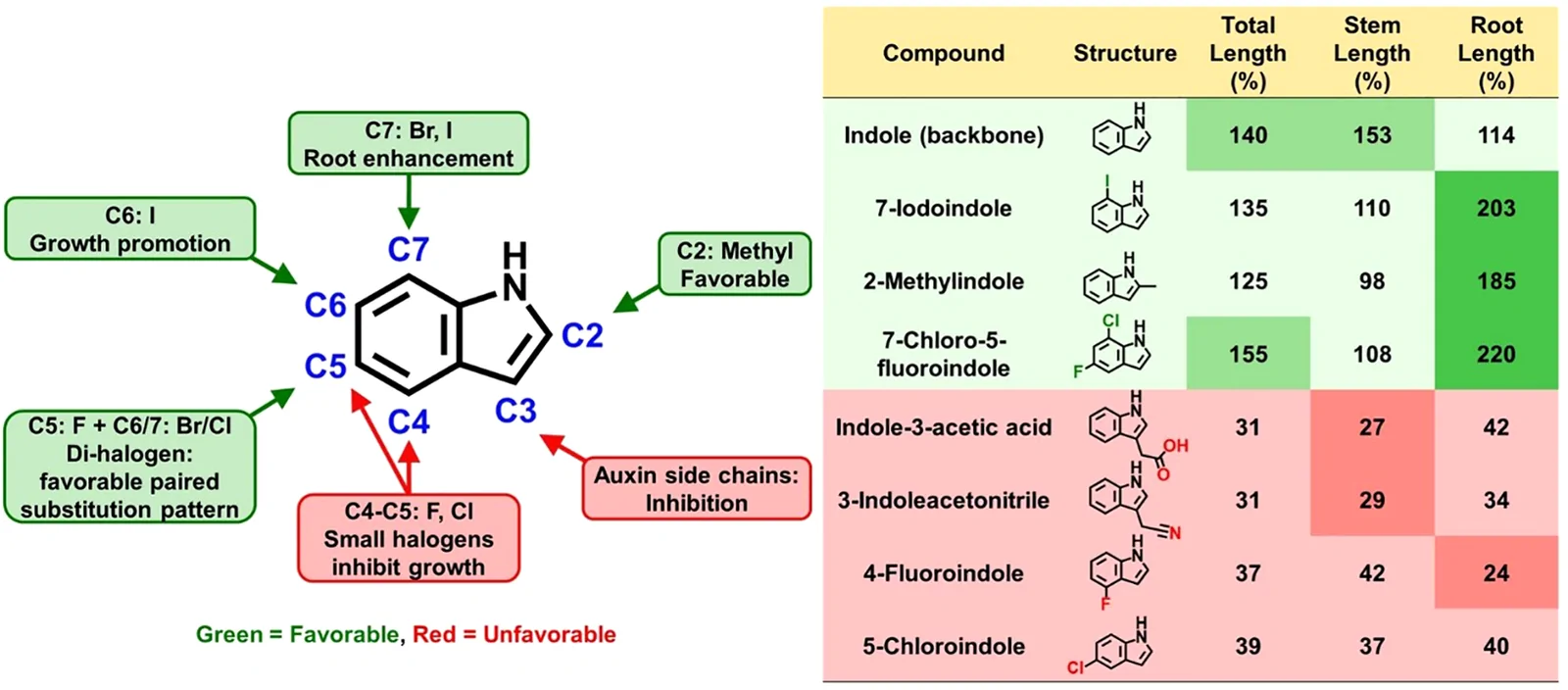
Structure–activity relationship of substituted indoles in radish seedlings.

Di-halogenated indoles further clarified position-dependent effects (**Figure 3**; **Supplementary Table 1**). Strong root-dominant growth promotion was observed when C5 was paired with substitutions at C6 or C7, particularly in combinations involving fluorine at C5 with bromine or chlorine at C6 or C7. The most prominent example was 7-chloro-5-fluoroindole (~155% total; root ~220%), followed by 7-bromo-5-fluoroindole (~142% total; root ~211%) and 6-bromo-5-fluoroindole (~142% total; root ~180%), indicating that C5/C6–C7 paired substitution is a favorable motif for root enhancement. A second favorable pattern was observed with C4/C6 di-substitution, where 4,6-dibromoindole (~137% total; root ~198%) and 4-bromo-6-chloroindole (~128% total; root ~197%) also produced strong root-biased phenotypes. However, not all halogen pairs were beneficial; multi-fluorinated substitutions and certain mixed combinations were frequently associated with reduced growth, suggesting that excessive electron-withdrawing substitution can negatively impact activity.

Non-halogen substitutions also influenced plant growth (**Figure 3**; **Supplementary Table 1**). 2-methyl substitution was consistently favorable and produced a root-dominant phenotype (~125% total; root ~185%), while 2-methylindole-3-carboxaldehyde (~142% total; root ~170%) further supported the importance of substitutions near the C2 position. Overall, the SAR indicates that plant growth promotion is favored by substitutions at C6–C7, particularly with larger halogens (Br, I), and by certain paired substitutions involving C5, whereas substitutions at C4–C5 with smaller halogens (F, Cl) and auxin-like side chains are generally unfavorable.

## 4 Discussion

This study shows that indole and halogenated indoles can strongly modulate early seedling growth, with the most pronounced effects observed in root development. The parent scaffold indole promoted growth at 10 μg/mL, whereas classical auxin-related derivatives such as IAA and IAN suppressed growth under the same conditions (**Figure 2**). Halogen substitution further enhanced activity, and several di-halogenated indoles produced the strongest root-biased phenotypes. These responses were consistently observed in both radish and Chinese cabbage, indicating conserved growth modulation across *Brassicaceae* species (**Figure 2**).

Auxin responses are highly concentration-dependent. Previous studies have consistently shown that low concentrations of IAA promote root growth and development by stimulating cell elongation and lateral root formation, whereas excessive auxin levels suppress primary root elongation through disruption of auxin homeostasis and signaling (Roychoudhry and Kepinski, 2022; Zhao, 2010). Therefore, the inhibitory effects of IAA and IAN observed at 10 μg/mL in the present study are consistent with this established concentration-dependent auxin response rather than contradicting the well-known growth-promoting role of auxin at lower concentrations. Furthermore, the use of a moderately high screening concentration facilitated the identification of compounds that retained robust growth-modulating activity under relatively stringent screening conditions, rather than compounds exhibiting effects only within a narrow low-dose range. In contrast, indole strongly promoted seedling growth, suggesting that it does not act simply as an auxin precursor in this system. Indole is recognized as a signaling molecule in plant–microbe interactions and can interfere with auxin transport and signaling, thereby reshaping root development (Bailly et al., 2014; Lee et al., 2015). The strong growth-promoting activity observed here supports a role for indole as a non-canonical regulator of plant growth.

The growth-promoting effect of indole, particularly on stem elongation, may arise from modulation of endogenous hormone balance rather than direct auxin mimicry (Bartel, 1997; Normanly, 2006). Although indole is structurally related to IAA, its promotion of stem growth—contrasting with the strong inhibition observed for IAA and IAN at the same concentration—suggests that indole does not simply elevate auxin signaling. Instead, indole may subtly influence auxin homeostasis by enhancing local IAA biosynthesis at physiologically moderate levels or by altering auxin transport dynamics, thereby avoiding the supra-optimal auxin accumulation that suppresses elongation (Normanly, 2006, 2010). Indole has been reported to interfere with polar auxin transport and PIN transporter activity (Bailly et al., 2014), which could redistribute endogenous auxin toward elongation zones in stems and hypocotyls.

In addition, stem elongation is strongly regulated by auxin–gibberellin (GA) cross-talk (Fu and Harberd, 2003; Weiss and Ori, 2007). A moderate increase in auxin signaling can stimulate GA biosynthesis, leading to DELLA degradation and enhanced cell expansion (Fu and Harberd, 2003). Thus, indole may act upstream of GA-dependent elongation pathways. Another plausible mechanism involves modulation of cell wall extensibility; indole exposure could influence the expression of expansins or proton pump activity, indirectly promoting cell elongation (Blatt, 2000; Morsomme and Boutry, 2000).

In addition, physicochemical properties of the parent indole scaffold, including hydrophobicity and molecular flexibility, may influence cellular uptake and biological activity. In contrast, classical auxin derivatives bearing side chains directly engage the TIR1/AFB pathway (Dharmasiri et al., 2005; Tan et al., 2007), leading to dose-dependent inhibition of primary growth at 10 μg/mL. Therefore, indole likely functions as a mild signaling modulator that enhances elongation growth through hormone-network fine-tuning rather than through canonical auxin receptor activation.

Besides gibberellin, auxin also interacts extensively with other phytohormones involved in seedling development. Auxin–cytokinin interactions coordinate the balance between cell division and differentiation (Schaller et al. 2015), whereas auxin–ethylene crosstalk regulates root elongation and adaptive growth responses (Stepanova et al., 2007; Wilkinson et al., 2012). Brassinosteroids further act synergistically with auxin to promote cell expansion and elongation (Nemhauser et al., 2004). Therefore, the distinct growth responses observed among indole derivatives may involve broader modulation of interconnected phytohormone signaling networks rather than auxin signaling alone.

Halogen substitution markedly altered the biological activity of the indole scaffold (**Figure 3**; **Supplementary Table 1**). Several di-halogenated indoles showed the strongest growth-promoting phenotypes, particularly through enhanced root elongation (**Figure 2**). Halogenation is known to influence molecular stability and bioavailability, and in auxin biology, halogenated derivatives such as 4-chloroindole-3-acetic acid exhibit altered potency and metabolism compared with IAA (Karcz and Burdach, 2002; Tivendale et al., 2012). Our results extend this concept to indoles lacking the auxin side chain and indicate that halogenation can be used to tune growth-modulating activity.

SAR analysis revealed clear position- and halogen-dependent trends (**Figure 3**). Substitutions at C6 and C7, particularly with bromine or iodine, were frequently associated with enhanced growth, while substitutions at C4 and C5 with smaller halogens were often unfavorable. Certain di-halogenation patterns produced the strongest root-dominant phenotypes, highlighting the importance of spatial arrangement on the indole ring. Across the compound library, root elongation was the most responsive trait, suggesting that root tissues are particularly sensitive to indole-based growth modulation (Friml and Jones, 2010; Malamy and Benfey, 1997). Given the central role of root architecture in nutrient uptake and early plant establishment, these effects are likely to have biological significance.

Recent studies consistently indicate that halogenation enhances the biological potency of the indole scaffold across multiple systems (Faleye et al., 2023). Multi-halogenated indoles with substitutions around the C4–C6 region have shown strong antibacterial, antifungal, and antibiofilm activities, often associated with increased membrane interactions, ROS generation, and suppression of virulence-related pathways (Jeong et al., 2025; Sim et al., 2025). These cross-kingdom activities suggest that halogen atoms can improve bioactivity by modulating physicochemical properties such as lipophilicity, binding affinity, and stability (Meanwell, 2011). Moreover, recent machine learning–based toxicity analyses indicate that halogen substitution itself does not necessarily increase toxicity, with scaffold features playing a more dominant role in determining safety profiles (Boya et al., 2025). Together, these observations support the concept that halogenation functions as a general chemical lever to enhance indole bioactivity, consistent with the strong root-growth modulation observed in the present plant system.

The strong growth suppression observed with IAN and several halogenated indoles suggests potential applicability as plant growth regulators or herbicide-like agents. Synthetic auxin herbicides such as 2,4-dichlorophenoxyacetic acid (Song, 2014) and dicamba (Behrens et al., 2007) exploit dysregulated auxin signaling to induce uncontrolled growth, vascular disruption, and eventual plant death. In our assay, IAA and IAN markedly inhibited seedling elongation at 10 μg/mL (**Figure 2**), consistent with supra-optimal auxin activity. Although IAN itself is a natural indole derivative in plants, its inhibitory phenotype indicates that indole-based scaffolds lacking optimal substitution patterns can disrupt early growth processes. Furthermore, specific halogenation patterns were also associated with strong growth suppression, suggesting that structural tuning of the indole nucleus can shift activity from growth promotion to inhibition. Given that halogen substitution often enhances metabolic stability and bioavailability (Meanwell, 2011), selected halogenated indoles may serve as lead scaffolds for developing novel indole-based plant growth inhibitors or herbicidal agents. Future studies evaluating dose–response behavior, selectivity among crop and weed species, and molecular engagement of the TIR1/AFB pathway would clarify their translational potential.

The growth-modulating effects of representative compounds were reproducible in both radish and Chinese cabbage, indicating that these responses are not species-specific but reflect conserved physiological processes within *Brassicaceae* seedlings. As a discovery-focused study, the underlying molecular mechanisms remain to be clarified, and longer-term effects on plant development were not assessed.

## 5 Conclusion

In conclusion, screening of 87 indole derivatives demonstrated that while seed germination was largely unaffected, plant growth responses, particularly root development, varied markedly depending on the substitution pattern. Among the tested compounds, indole showed strong overall seedling growth promotion, whereas 7-chloro-5-fluoroindole exhibited the strongest root growth-promoting activity. In contrast, IAA and IAN showed dose-dependent growth-inhibitory effects. Structure–activity relationship analysis further demonstrated that the identity and position of halogen substitution were key determinants of growth-modulating activity. These findings establish halogenated indoles as promising modulators of early plant growth and provide a framework for the rational design of indole-based plant growth regulators.

## Data Availability

The original contributions presented in the study are included in the article/**Supplementary Material**. Further inquiries can be directed to the corresponding author.
