# Supplementary material for: Halogenated indoles selectively modulate plant growth and root development
Source: Front Plant Sci. 2026 Sep 15;17:1826917. doi: 10.3389/fpls.2026.1826917 (PMC13619941; doi:10.3389/fpls.2026.1826917)
Supplement: Supplementary file 1 [file Table1.docx]

Supplementary Material

Halogenated indoles selectively modulate plant growth and root development

Hyeonwoo Jeong, Kwang-Hyun Baek, Jin-Hyung Lee^*^, Jintae Lee^*^

School of Chemical Engineering, Yeungnam University, Gyeongsan, 38541, Republic of Korea; [wawdgud@yu.ac.kr](mailto:wawdgud@yu.ac.kr) (H.J.)

Department of Biotechnology, Yeungnam University, Gyeongsan, 38541, Republic of Korea; khbaek@ynu.ac.kr (K.-H.B.)

*** Correspondence:**
[jinhlee@ynu.ac.kr](mailto:jinhlee@ynu.ac.kr) (J.-H.L.); jtlee@ynu.ac.kr (J.L.); Tel.: +82-53-810-3812 (J.-H.L.); Tel.: +82-53-810-2533 (J.L.)

# 1 Supplementary Table~~s~~

**Supplementary Table 1. Chemical structures and growth responses of 87 indole derivatives** **at 10 μg/mL in radish seedlings for 5 days.** Growth values are expressed relative to the untreated control (set to 100%).

| **No.** | **Name** | **CAS Number** | **Purity (%)** | **Structure** | **Total growth rate (%)** | **Stem growth rate (%)** | **Root growth rate (%)** |
| --- | --- | --- | --- | --- | --- | --- | --- |
| 1* | Indole | 120-72-9 | 98 |  | 140 ± 20 | 153 ± 24 | 114 ± 33 |
| 2 | Indole-3-acetamide | 879-37-8 | 98 |  | 104 ± 24 | 108 ± 26 | 94 ± 21 |
| 3* | Indole-3-acetic acid | 87-51-4 | 98 |  | 31 ± 8 | 27 ± 8 | 42 ± 12 |
| 4 | Indole-3-butyric acid | 133-32-4 | 98 |  | 34 ± 11 | 27 ± 12 | 50 ± 16 |
| 5 | Indole-3-pyruvic acid | 392-12-1 | 97 |  | 59 ± 23 | 61 ± 26 | 53 ± 24 |
| 6 | 1-Acetyl-2,3-dihydroindole-5-sulfonyl chloride | 52206-05-0 | 97 |  | 86 ± 47 | 80 ± 48 | 99 ± 59 |
| 7 | 1-BOC-7-methylindole | 442910-62-5 | 98 |  | 126 ± 17 | 128 ± 20 | 122 ± 31 |
| 8 | Indole-2-carbaldehyde | 19005-93-7 | 98 |  | 91 ± 35 | 91 ± 39 | 90 ± 35 |
| 9 | 2-Methylindole | 95-20-5 | 98 |  | 125 ± 60 | 98 ± 38 | 185 ± 114 |
| 10 | 2-Methylindole-3-acetic acid | 1912-43-2 | 98 |  | 38 ± 20 | 39 ± 26 | 37 ± 16 |
| 11 | 2-Methylindole-3-carboxaldehyde | 5416-80-8 | 96 |  | 142 ± 63 | 125 ± 50 | 170 ± 93 |
| 12 | 2,3-Dimethylindole | 91-55-4 | 98 |  | 103 ± 31 | 101 ± 38 | 107 ± 35 |
| 13 | 3-(2-Hydroxyethyl)indole | 526-55-6 | 98 |  | 90 ± 27 | 91 ± 28 | 86 ± 32 |
| 14* | 3-Indoleacetonitrile | 771-51-7 | 98 |  | 31 ± 10 | 29 ± 9 | 34 ± 13 |
| 15 | 3-Methylindole | 83-34-1 | 98 |  | 100 ± 31 | 108 ± 44 | 88 ± 26 |
| 16 | 4-Bromo-5,7-difluoroindole | 1381878-59-6 | 98 |  | 34 ± 7 | 26 ± 7 | 50 ± 16 |
| 17 | 4-Bromo-5-fluoroindole | 1227493-96-0 | 98 |  | 50 ± 21 | 47 ± 22 | 55 ± 22 |
| 18 | 4-Bromo-6-chloroindole | 885519-23-3 | 98 |  | 128 ± 36 | 102 ± 25 | 197 ± 83 |
| 19 | 4-Bromo-6-fluoroindole | 885520-70-7 | 97 |  | 91 ± 32 | 83 ± 32 | 108 ± 43 |
| 20 | 4-Bromo-7-chloroindole | 126811-30-1 | 97 |  | 112 ± 30 | 95 ± 30 | 156 ± 48 |
| 21 | 4-Bromo-7-fluoroindole | 883500-66-1 | 96 |  | 82 ± 35 | 67 ± 32 | 114 ± 54 |
| 22 | 4-Bromoindole | 52488-36-5 | 97 |  | 105 ± 16 | 97 ± 13 | 119 ± 35 |
| 23 | 4-Chloroindole | 25235-85-2 | 97 |  | 55 ± 13 | 35 ± 20 | 66 ± 19 |
| 24 | 4-Fluoroindole | 387-43-9 | 98 |  | 37 ± 6 | 42 ± 15 | 24 ± 8 |
| 25 | 4-Iodoindole | 81038-38-2 | 98 |  | 79 ± 22 | 78 ± 28 | 79 ± 23 |
| 26 | 4-Methylindole | 16096-32-5 | 96 |  | 75 ± 16 | 101 ± 26 | 32 ± 12 |
| 27 | 4,5-Dichloroindole | 122509-73-3 | 98 |  | 105 ± 39 | 96 ± 34 | 126 ± 58 |
| 28 | 4,6-Dibromoindole | 99910-50-6 | 95 |  | 137 ± 25 | 114 ± 22 | 198 ± 70 |
| 29 | 4,6-Dichloroindole | 101495-18-5 | 97 |  | 52 ± 37 | 44 ± 31 | 67 ± 60 |
| 30 | 4,6-Dichloroindole-2,3-dione | 18711-15-4 | 95 |  | 79 ± 54 | 73 ± 59 | 92 ± 51 |
| 31 | 4,6-Difluoroindole | 199526-97-1 | 98 |  | 67 ± 34 | 74 ± 40 | 53 ± 29 |
| 32 | 4,7-Dibromoindole | 126811-31-2 | 97 |  | 91 ± 41 | 71 ± 40 | 135 ± 70 |
| 33 | 4,7-Dichloroisatin | 18711-13-2 | 97 |  | 122 ± 63 | 101 ± 60 | 133 ± 68 |
| 34 | 5-(Trifluoromethoxy)Indolin-2,3-dione | 169037-23-4 | 97 |  | 127 ± 19 | 131 ± 30 | 121 ± 22 |
| 35 | 5-Amino-2-Methylindole | 7570-49-2 | 95 |  | 79 ± 13 | 72 ± 18 | 95 ± 29 |
| 36 | 5-Bromo-4-chloroindole | 217656-69-4 | 98 |  | 104 ± 28 | 91 ± 30 | 138 ± 41 |
| 37 | 5-Bromo-4-fluoroindole | 344790-96-1 | 95 |  | 81 ± 36 | 82 ± 40 | 80 ± 36 |
| 38 | 5-Bromo-6-chloroindole | 122531-09-3 | 97 |  | 100 ± 36 | 94 ± 39 | 113 ± 40 |
| 39 | 5-Bromo-6-chloro-2,3-dihydroindole | 1368146-95-5 | 95 |  | 58 ± 39 | 48 ± 36 | 79 ± 47 |
| 40 | 5-Bromo-6-fluoroindole | 434960-42-6 | 96 |  | 112 ± 34 | 123 ± 43 | 90 ± 32 |
| 41 | 5-Bromo-7-chloroindole | 180623-89-6 | 97 |  | 94 ± 46 | 82 ± 38 | 120 ± 78 |
| 42 | 5-Bromo-7-fluoroindole | 883500-73-0 | 95 |  | 33 ± 16 | 30 ± 17 | 39 ± 19 |
| 43 | 5-Bromoindole | 10075-50-0 | 98 |  | 69 ± 15 | 74 ± 23 | 59 ± 17 |
| 44 | 5-Chloro-6-fluoroindole | 169674-57-1 | 97 |  | 111 ± 57 | 106 ± 56 | 121 ± 77 |
| 45 | 5-Chloroindole | 17422-32-1 | 98 |  | 39 ± 15 | 37 ± 8 | 40 ± 22 |
| 46 | 5-Fluoro-2-methylindole | 399-72-4 | 95 |  | 122 ± 47 | 103 ±35 | 151 ± 76 |
| 47 | 5-Fluoroindole | 399-52-0 | 98 |  | 33 ± 9 | 37 ± 14 | 24 ± 5 |
| 48 | 5-Formylindole | 1196-69-6 | 98 |  | 65 ± 29 | 66 ± 30 | 63 ± 27 |
| 49 | 5-Iodoindole | 16066-91-4 | 98 |  | 97 ± 14 | 96 ± 17 | 100 ± 26 |
| 50 | 5-Methylindole | 614-96-0 | 98 |  | 47 ± 15 | 46 ± 22 | 48 ± 11 |
| 51 | 5,6-Dibromoindole | 854923-38-9 | 96 |  | 101 ± 28 | 93 ± 27 | 119 ± 40 |
| 52 | 5,6-Dichloroindole-2,3-dione | 1677-48-1 | 97 |  | 76 ± 41 | 74 ± 40 | 82 ± 46 |
| 53 | 5,6-Dichloroindole | 121859-57-2 | 98 |  | 103 ± 52 | 92 ± 40 | 128 ± 93 |
| 54 | 5,6-Dichloroindolin-2-one | 71293-59-9 | 97 |  | 69 ± 49 | 82 ± 41 | 68 ± 55 |
| 55 | 5,6-Difluoroindole | 169674-01-5 | 97 |  | 54 ± 19 | 62 ± 30 | 39 ± 10 |
| 56 | 5,7-Dibromoindole | 36132-08-8 | 95 |  | 79 ± 30 | 66 ± 29 | 105 ± 43 |
| 57 | 5,7-Dibromoindole-2,3-dione | 6374-91-0 | 98 |  | 133 ± 34 | 140 ± 34 | 118 ± 53 |
| 58 | 5,7-Dichloroindole | 4792-72-7 | 97 |  | 95 ± 36 | 78 ± 31 | 129 ± 62 |
| 59 | 6-Benzyloxyindole | 15903-94-3 | 97 |  | 57 ± 25 | 51 ± 22 | 70 ± 39 |
| 60 | 6-Bromo-4-fluoroindole | 885520-59-2 | 98 |  | 88 ± 42 | 82 ± 39 | 100 ± 80 |
| 61 | 6-Bromo-4-iodoindole | 885519-17-5 | 98 |  | 126 ± 21 | 108 ± 24 | 175 ± 44 |
| 62 | 6-Bromo-5-chloroindole | 1191028-50-8 | 95 |  | 64 ± 43 | 53 ± 40 | 87 ± 58 |
| 63 | 6-Bromo-5-fluoroindole | 259860-08-7 | 96 |  | 142 ± 41 | 125 ± 35 | 180 ± 72 |
| 64 | 6-Bromoindole | 52415-29-9 | 97 |  | 109 ± 31 | 104 ± 33 | 119 ± 46 |
| 65 | 6-Chloro-5-fluoroindole | 122509-72-2 | 97 |  | 121 ± 57 | 119 ± 54 | 124 ± 75 |
| 66 | 6-Chloro-7-fluoroindole | 259860-04-3 | 98 |  | 59 ± 37 | 57 ± 38 | 61 ± 42 |
| 67 | 6-Chloroindole | 17422-33-2 | 97 |  | 39 ± 14 | 50 ± 19 | 19 ± 2 |
| 68 | 6-Fluoro-4-iodoindole | 885520-49-0 | 95 |  | 82 ± 40 | 70 ± 39 | 106 ±59 |
| 69 | 6-Fluoro-5-iodoindole | 908600-77-1 | 95 |  | 78 ± 55 | 69 ± 50 | 97 ± 74 |
| 70 | 6-Fluoroindole | 399-51-9 | 98 |  | 46 ± 11 | 22 ± 5 | 58 ± 16 |
| 71 | 6-Iodo-2,3-dihydroindole | 114144-16-0 | 96 |  | 95 ± 31 | 92 ± 29 | 103 ± 47 |
| 72 | 6-Iodoindole | 115666-47-2 | 98 |  | 130 ± 41 | 126 ± 34 | 139 ± 70 |
| 73 | 7-Bromo-4-chloroindole | 126811-29-8 | 95 |  | 78 ± 39 | 68 ± 33 | 102 ± 55 |
| 74 | 7-Bromo-4-fluoroindole | 292636-09-0 | 98 |  | 112 ± 54 | 103 ± 52 | 132 ± 73 |
| 75 | 7-Bromo-5-fluoroindole | 408355-23-7 | 97 |  | 142 ± 61 | 110 ± 49 | 211 ± 102 |
| 76 | 7-Bromo-5-chloroindole | 292636-08-9 | 97 |  | 74 ± 35 | 61 ± 30 | 101 ± 63 |
| 77 | 7-Bromoindole | 51417-51-7 | 97 |  | 117 ± 37 | 83 ± 32 | 182 ± 61 |
| 78* | 7-Chloro-5-fluoroindole | 259860-01-0 | 95 |  | 155 ± 61 | 108 ± 40 | 220 ± 122 |
| 79 | 7-Chloroindole | 53924-05-3 | 98 |  | 96 ± 37 | 76 ± 33 | 133 ± 68 |
| 80 | 7-Fluoro-5-iodoindole | 1173023-15-8 | 97 |  | 46 ± 15 | 40 ± 16 | 60 ± 19 |
| 81 | 7-Fluoroindole | 387-44-0 | 97 |  | 59 ± 11 | 71 ± 17 | 34 ± 8 |
| 82 | 7-Iodoindole | 89976-15-8 | 95 |  | 135 ± 28 | 110 ± 29 | 203 ±47 |
| 83 | 7-Methylindole | 933-67-5 | 98 |  | 117 ± 47 | 103 ± 48 | 139 ± 55 |
| 84 | 7-Methylindole-3-carboxaldehyde | 4771-50-0 | 96 |  | 139 ± 24 | 138 ± 25 | 140 ± 41 |
| 85 | 7-Methylindole-2,3-dione | 1127-59-9 | 97 |  | 118 ± 49 | 123 ± 57 | 110 ± 50 |
| 86 | 7-Nitroindole | 6960-42-5 | 98 |  | 84 ± 34 | 70 ± 36 | 116 ± 49 |
| 87 | 8-Fluoroquinoline | 394-68-3 | 95 |  | 71 ± 33 | 70 ± 34 | 73 ± 30 |

* Four distinctive compounds are indicated in red.
